# A component overlapping attribute clustering (COAC) algorithm for single-cell RNA sequencing data analysis and potential pathobiological implications

**DOI:** 10.1371/journal.pcbi.1006772

**Published:** 2019-02-19

**Authors:** He Peng, Xiangxiang Zeng, Yadi Zhou, Defu Zhang, Ruth Nussinov, Feixiong Cheng

**Affiliations:** 1 Department of Computer Science, Xiamen University, Xiamen, Fujian, China; 2 Department of Chemistry and Biochemistry, Ohio University, Athens, OH, United States of America; 3 Cancer and Inflammation Program, Leidos Biomedical Research, Inc., Frederick National Laboratory for Cancer Research, National Cancer Institute at Frederick, Frederick, MD, United States of America; 4 Department of Human Molecular Genetics and Biochemistry, Sackler School of Medicine, Tel Aviv University, Tel Aviv, Israel; 5 Genomic Medicine Institute, Lerner Research Institute, Cleveland Clinic, Cleveland, OH, United States of America; 6 Department of Molecular Medicine, Cleveland Clinic Lerner College of Medicine, Case Western Reserve University, Cleveland, OH, United States of America; 7 Case Comprehensive Cancer Center, Case Western Reserve University School of Medicine, Cleveland, OH, United States of America; National Institutes of Health, UNITED STATES

## Abstract

Recent advances in next-generation sequencing and computational technologies have enabled routine analysis of large-scale single-cell ribonucleic acid sequencing (scRNA-seq) data. However, scRNA-seq technologies have suffered from several technical challenges, including low mean expression levels in most genes and higher frequencies of missing data than bulk population sequencing technologies. Identifying functional gene sets and their regulatory networks that link specific cell types to human diseases and therapeutics from scRNA-seq profiles are daunting tasks. In this study, we developed a Component Overlapping Attribute Clustering (COAC) algorithm to perform the localized (cell subpopulation) gene co-expression network analysis from large-scale scRNA-seq profiles. Gene subnetworks that represent specific gene co-expression patterns are inferred from the components of a decomposed matrix of scRNA-seq profiles. We showed that single-cell gene subnetworks identified by COAC from multiple time points within cell phases can be used for cell type identification with high accuracy (83%). In addition, COAC-inferred subnetworks from melanoma patients’ scRNA-seq profiles are highly correlated with survival rate from The Cancer Genome Atlas (TCGA). Moreover, the localized gene subnetworks identified by COAC from individual patients’ scRNA-seq data can be used as pharmacogenomics biomarkers to predict drug responses (The area under the receiver operating characteristic curves ranges from 0.728 to 0.783) in cancer cell lines from the Genomics of Drug Sensitivity in Cancer (GDSC) database. In summary, COAC offers a powerful tool to identify potential network-based diagnostic and pharmacogenomics biomarkers from large-scale scRNA-seq profiles. COAC is freely available at https://github.com/ChengF-Lab/COAC.

## Author summary

Single-cell RNA sequencing (scRNA-seq) can reveal complex and rare cell populations, uncover gene regulatory relationships, track the trajectories of distinct cell lineages in development, and identify cell-cell variabilities in human diseases and therapeutics. Although experimental methods for scRNA-seq are increasingly accessible, computational approaches to infer gene regulatory networks from raw data remain limited. From a single-cell perspective, the stochastic features of a single cell must be properly embedded into gene regulatory networks. However, it is difficult to identify technical noise (e.g., low mean expression levels and missing data) and cell-cell variabilities remain poorly understood. In this study, we introduced a network-based approach, termed Component Overlapping Attribute Clustering (COAC), to infer novel gene-gene subnetworks in individual components (subsets of whole components) representing multiple cell types and phases of scRNA-seq data. We showed that COAC can reduce batch effects and identify specific cell types in two large-scale human scRNA-seq datasets. Importantly, we demonstrated that gene subnetworks identified by COAC from scRNA-seq profiles highly correlated with patients's survival and drug responses in cancer, offering a novel computational tool for advancing precision medicine.

## Introduction

Single cell ribonucleic acid sequencing (scRNA-seq) offers advantages for characterization of cell types and cell-cell heterogeneities by accounting for dynamic gene expression of each cell across biomedical disciplines, such as immunology and cancer research [1, 2]. Recent rapid technological advances have expanded considerably the single cell analysis community, such as The Human Cell Atlas (THCA) [3]. The single cell sequencing technology offers high-resolution cell-specific gene expression for potentially unraveling of the mechanism of individual cells. The THCA project aims to describe each human cell by the expression level of approximately 20,000 human protein-coding genes; however, the representation of each cell is high dimensional, and the human body has trillions of cells. Furthermore, scRNA-seq technologies have suffered from several limitations, including low mean expression levels in most genes and higher frequencies of missing data than bulk sequencing technology [4]. Development of novel computational technologies for routine analysis of scRNA-seq data are urgently needed for advancing precision medicine [5].

Inferring gene-gene relationships (e.g., regulatory networks) from large-scale scRNA-seq profiles is limited. Traditional approaches to gene co-expression network analysis are not suitable for scRNA-seq data due to a high degree of cell-cell variabilities. For example, LEAP (Lag-based Expression Association for Pseudotime-series) is an R package for constructing gene co-expression networks using different time points at the single cell level [6]. The Partial information decomposition (PID) algorithm aims to predict gene-gene regulatory relationships [7]. Although these computational approaches are designed to infer gene co-expression networks from scRNA-seq data, they suffer from low resolution at the single-cell or single-gene levels.

In this study, we introduced a network-based approach, termed Component Overlapping Attribute Clustering (COAC), to infer novel gene-gene subnetwork in individual components (the subset of whole components) representing multiple cell types and cell phases of scRNA-seq data. Each gene co-expression subnetwork represents the co-expressed relationship occurring in certain cells. The scoring function identifies co-expression networks by quantifying uncoordinated gene expression changes across the population of single cells. We showed that gene subnetworks identified by COAC from scRNA-seq profiles were highly correlated with the survival rate of melanoma patients and drug responses in cancer cell lines, indicating a potential pathobiological application of COAC. If broadly applied, COAC can offer a powerful tool for identifying gene-gene networks from large-scale scRNA-seq profiles in multiple diseases in the on-going development of precision medicine.

## Results

### Overview of Component Overlapping Attribute Clustering (COAC)

**Fig 1:**
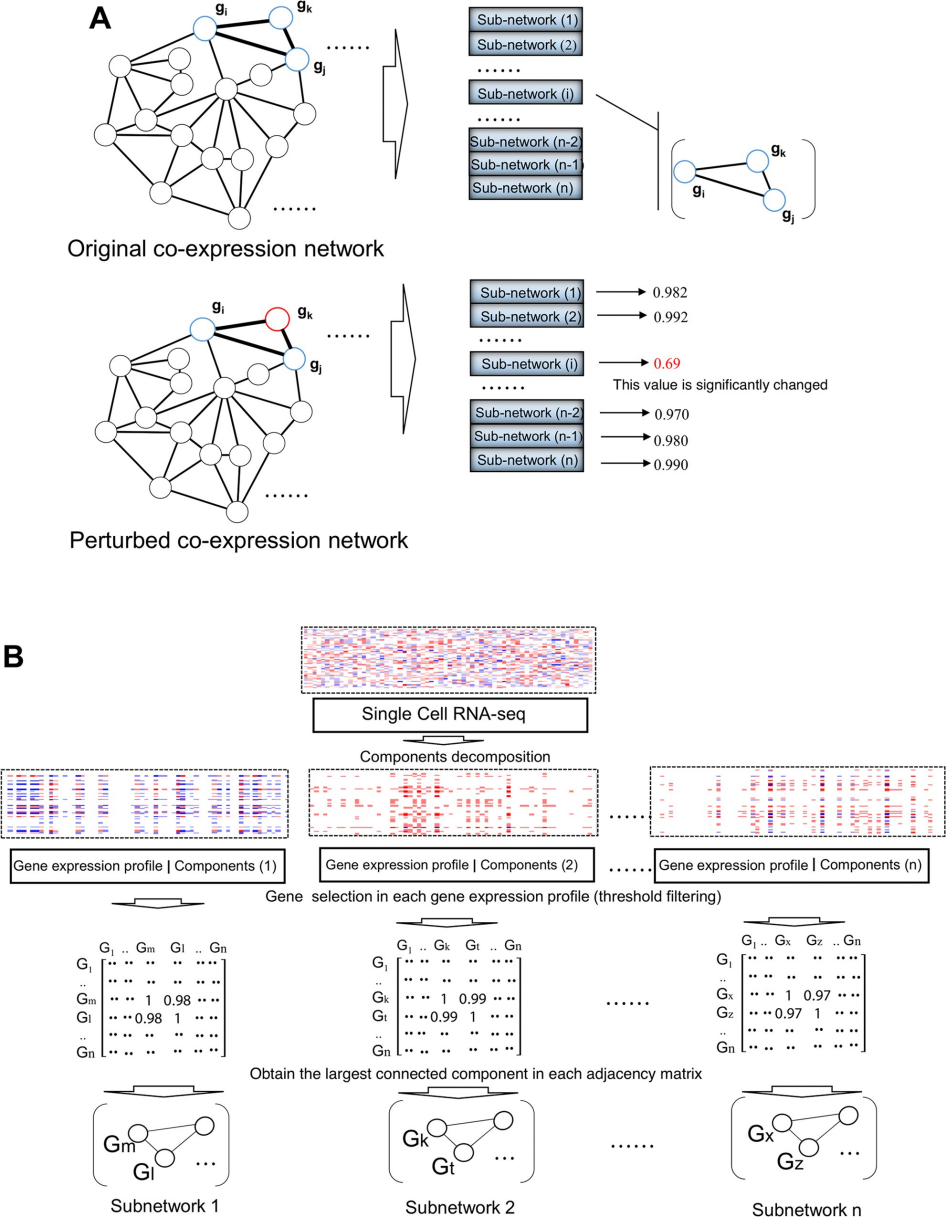
Diagram illustrating a Components Overlapping Attribute Clustering (COAC) algorithm for inferring gene-gene relationships from scRNA-seq data. (**A**) The whole gene co-expression network is decomposed into gene clusters (subnetworks). Each subnetwork is used to evaluate which degree of genes in the co-expression matrix derived from scRNA-seq data. If several genes express abnormally, the value of the subnetwork which contains those genes will change significantly. (**B**) The scRNA-seq data was decomposed into individual gene expression profile with specific components. After gene selection from each gene expression profile, the largest connected component was obtained as the subnetwork (see Methods).

In this study, we present a novel algorithm for inferring gene-gene networks from scRNA-seq data. Specifically, a gene-gene network represents the co-expression relationship of certain components (genes), which indicates the localized (cell subpopulation) co-expression from large-scale scRNA-seq profiles (**Fig 1**). Specifically, each gene subnetwork is represented by one or multiple feature vectors, which are learned from the scRNA-seq profile of the training set. For the test set, each gene expression profile can be transformed to a feature value by one or several feature vectors which measure the degree of coordination of gene co-expression. Since the feature vectors are learned from the relative expression of each gene, batch effects can be eliminated by normalization of relatively co-expressed genes (see Methods). In addition to showing that COAC can be used for batch effect elimination, we further validated COAC by illustrating three potential pathobiological applications: (1) cell type identification in two large-scale human scRNA-seq datasets (43,099 and 43,745 cells respectively, see Methods); (2) gene subnetworks identified from melanoma patients-derived scRNA-seq data showing high correlation with survival of melanoma patients from The Cancer Genome Atlas (TCGA); (3) gene subnetworks identified from scRNA-seq profiles which can be used to predict drug sensitivity/resistance in cancer cell lines.

### Batch effect elimination

**Fig 2:**
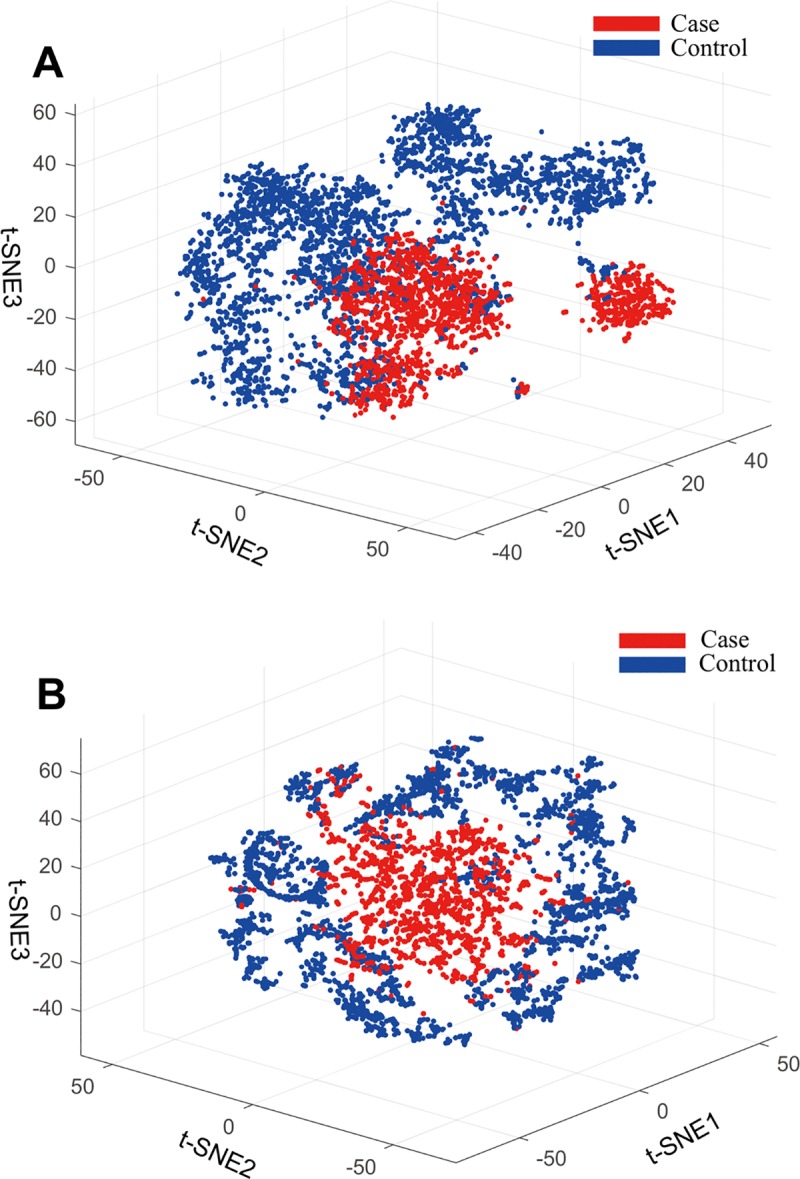
Batch effect elimination by COAC evaluated by a t-SNE algorithm [9]. (**A**) A significant batch effect elimination (Cells distribute separately in different groups) based on the COAC-inferred subnetworks. (**B**) A significant batch effect (Cells distribute uniformly between case and control groups) was observed based on the original scRNA-seq data from a previous study [37], without applying COAC.

We collected scRNA-seq data generated from 10x scRNA-seq protocol [7,8]. In total, 14,032 cells extracted from peripheral blood mononuclear cells (PBMC) in systemic lupus erythematosus (SLE) patients were used as the case group and 29,067 cells were used as the control group (see Methods). For the case group, we used 12,277 cells for the training set and the remaining 1,755 cells for the validation set. For the control group, we used 25,433 cells for the training set and 3,634 for the validation set. After filtering with *average correlation* and *average component ratio* thresholds (see Methods), we obtained 93,951 co-expression subnetworks (gene clusters with components) by COAC. We transformed these co-expression gene clusters to feature vectors. Features whose variance distribution was significantly different in the case group *versus* the control group were kept (see Methods). Using a t-SNE algorithm implemented in the R package-tsne [9], we found that the single cells (from the case group) which were retrieved directly from the patients can be more robustly separated from the control group cells (**Fig 2B**), comparing to the original data (**Fig 2A**) without applying COAC. Thus, the t-SNE analysis reveals that batch effects can be significantly reduced by COAC (**Fig 2**).

### Cell type identification

**Fig 3:**
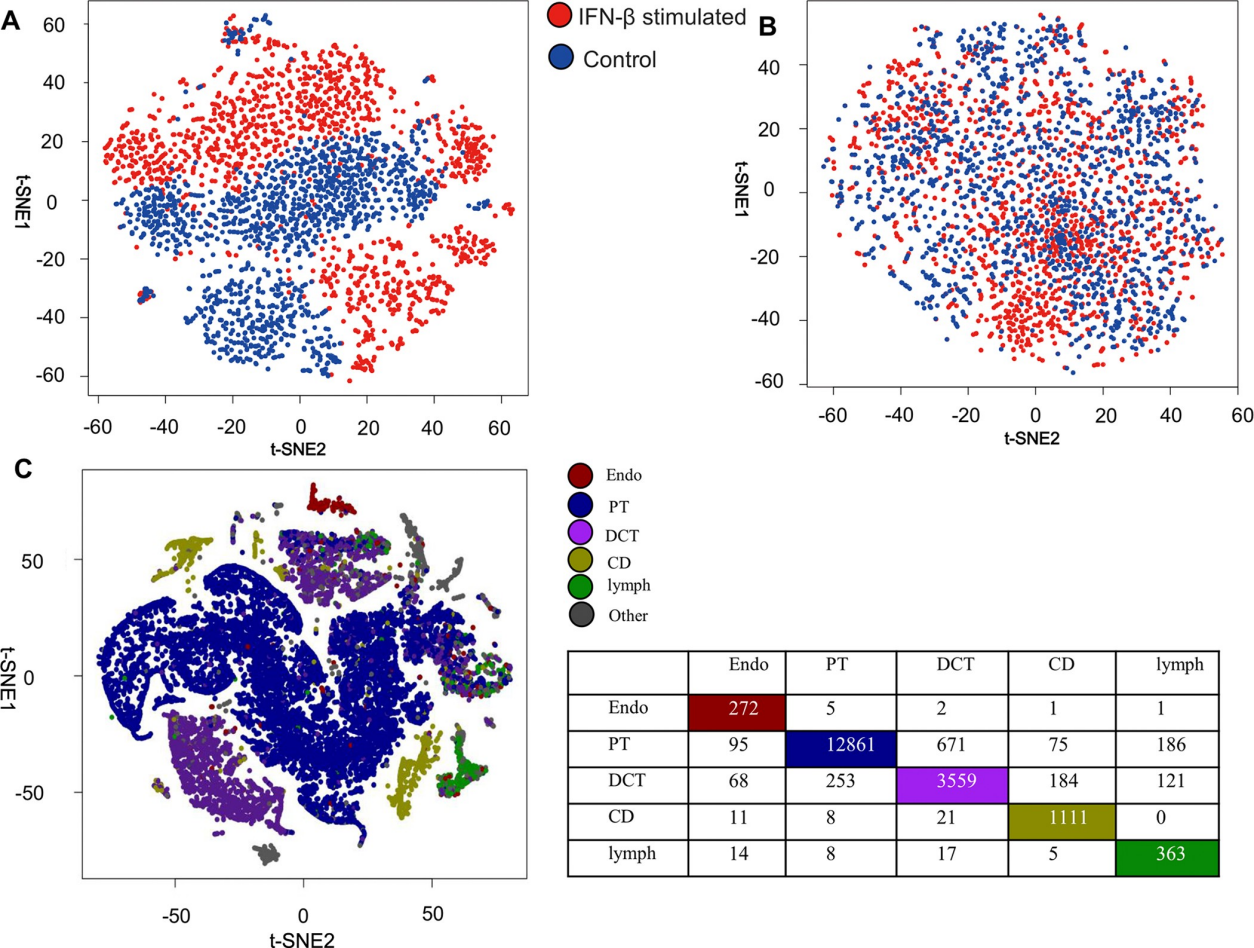
Accurate cell type identification by COAC. (**A**) The IFN-β stimulated and control groups are separated based on the subnetworks identified by COAC. (**B**) Cells from IFN-β stimulated and control groups are uniformly distributed in the whole space without applying COAC. (**C**) Five different cell types are identified with high accuracy based on gene subnetworks identified by COAC. Cell types for 83.05% cells have been identified correctly based on well-defined cell types from experimental data. Cell types are visualized by a t-SNE algorithm [9]. Endo: endothelial cells, PT: proximal tubule cells, DCT: distal convoluted tubule cells, CD: collecting duct principal cells, lymph: lymphocyte cells.

We next turned to examine whether COAC can be used for cell type identification. We collected a scRNA-seq dataset of 14,448 single cells in an IFN-β stimulated group and 14,621 single cells in the control group [8]. To remove factors caused by the stimulation conditions or experimental batch effects, we selected 13,003 cells in the IFN-β stimulated group and 13,158 cells in the control group as the training set to obtain homogeneous feature vectors for each cell. The remaining scRNA-seq data are used as the validation set. We generated the gene subnetworks by COAC and transformed the subnetworks into feature vectors for individual cells (see Methods). We found that cells from IFN-β stimulated and control groups were separated significantly (**Fig 3A**) by t-SNE [9]. However, without applying COAC cells from the IFN-β stimulated and control groups are uniformly distributed in the whole space (**Fig 3B**), suggesting that components which separate IFN-β stimulated cells from control cells were eliminated from the feature vector identified by COAC.

We further collected a scRNA-seq dataset including a total of 43,745 cells with well-defined cell types from a previous study [10]. We built a training set (21,873 cells) and a validation set (21,872 cells) with approximately equivalent size. In the training set, we generated co-expression subnetworks as the feature vector by COAC. For the validation set, we grouped the total cells into five main categories as described previously [10]. **Fig 3C** shows that COAC-inferred subnetworks can be used to distinguish five different cell types with high accuracy (cell types for 83.05% cells have been identified correctly) in the t-SNE analysis, indicating that COAC can identify cell types from heterogeneous scRNA-seq profiles. We next inspected potential pathobiological applications of COAC in identifying possible prognostic biomarkers or pharmacogenomics biomarkers in cancer.

### Network-based identification of prognostic biomarkers in melanoma

**Fig 4:**
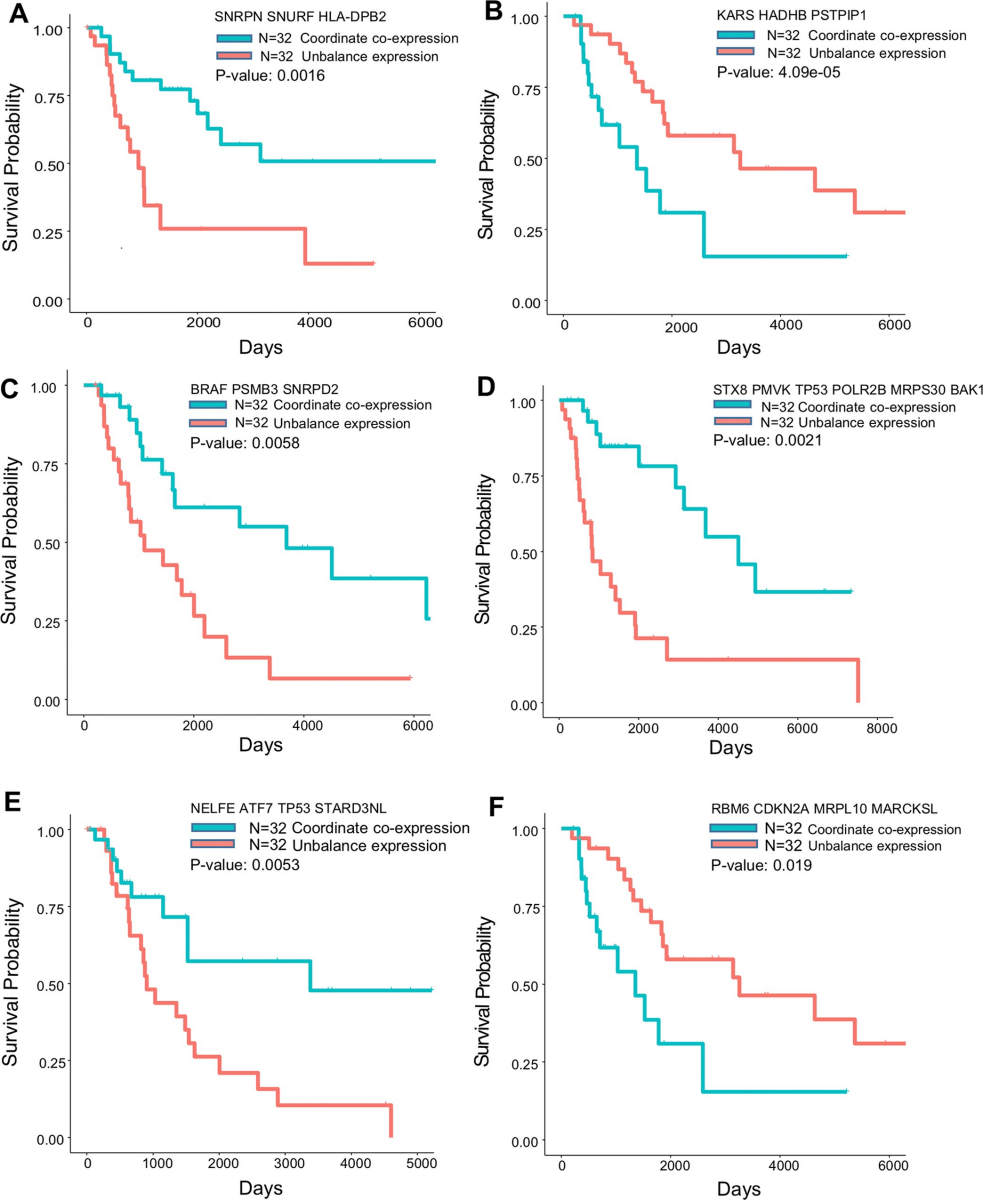
Survival analysis for COAC-inferred gene co-expression subnetworks in melanoma. (**A** and **B**) Survival analysis for COAC-inferred gene co-expression subnetworks from scRNA-seq data [11] by comparing malignant cells versus control cells from individual melanoma patients (see Methods). (**C** to **F**) Survival analysis for COAC-predicted gene subnetworks from scRNA-seq data by comparing T cells versus controls cells extracted from individual melanoma patients [11]. The top significantly selected subnetwork for each survival analysis was highlighted in each subfigure. The bulk RNA-seq data and clinical profiles for each melanoma patients were collected from TCGA website [13]. Survival analysis was conducted for these two groups using the R survival package [36] (see Methods).

We next turned to inspect whether COAC-inferred gene co-expression subnetworks can be used as potential prognostic biomarkers in clinical samples. We identified gene subnetworks from scRNA-seq data of melanoma patients [11]. Using a feature selection pipeline, we filtered the original subnetworks according to the difference of means and variances between two different groups (e.g., malignant cells versus control cells) to prioritize top gene co-expression subnetworks (**S1A Fig**). We collected the bulk gene expression data and clinical data for 458 melanoma patients from the TCGA website [12]. Applying COAC, we identified two gene co-expression subnetworks with the highest co-expression correlation in malignant cells compared to control cells (**S1B Fig**). For each subnetwork, we then calculated the co-expression correlation in bulk RNA-seq profiles of melanoma patients. Using the rank of co-expression values of melanoma patients, the top 32 patients were selected as group 1 and the tail 32 patients were selected as group 2. Log rank test was employed to compare the survival rate of two groups [13]. We found that gene subnetworks identified by COAC from melanoma patients-derived scRNA-seq data can predict patient survival rate (**Fig 4A** and **Fig 4B**). *KRAS*, is an oncogene in multiple cancer types [14], including menaloma [15]. Herein we found a co-expression among *KRAS*, *HADHB*, and *PSTPIP1*, can predict significantly patient survival rate (P-value = 4.09×10^−5^, log rank test, **Fig 4B**). Thus, regulation of KRAS-HADHB-PSTPIP1 may offer new a pathobiological pathway and potential biomarkers for predicting patient’s survival in menaloma.

We next focused on gene co-expression subnetworks in several known melanoma-related pathways, such as the MAPK, cell-cycle, DNA damage response, and cell death pathways [16] by comparing the differences in means and variances between T cell and other cells using COAC (see Methods). For each gene co-expression subnetwork identified by COAC, we selected 32 patients who had enriched co-expression correlation and 32 patients who had lost a co-expression pattern. We found that multiple COAC-inferred gene subnetworks predicted significantly menaloma patient survival rate (**Fig 4C–4F**). For example, we found that BRAF-PSMB3-SNRPD2 predict significant survival (P-value = 0.0058, log rank test. **Fig 4C**), revealing new potential disease pathways for *BRAF* melanoma. *CDKN2A*, encoding cyclin-dependent kinase Inhibitor 2A, plays important roles in melanoma [17]. Herein we found a potential regulatory subnetwork, RBM6-CDKN2A-MRPL10-MARCKSL, which is highly correlated with melanoma patients’ survival rate (P-value = 0.019, log rank test. **Fig 4F**). We identified several new potential regulatory subnetworks for *TP53* as well, which is highly correlated with patient's survival rate as well (**Fig 4D** and **4E**). Multiple novel COAC-inferred gene co-expression subnetworks that are significantly associated with patient’s survival rate are provided in **S2 Fig**.

Altogether, gene regulatory subnetworks identified by COAC can shed light on new disease mechanisms uncovering possible functional consequences of known melanoma genes and offer potential prognostic biomarkers in melanoma. COAC-inferred prognostic subnetworks should be further validated in multiple independent cohorts before clinical application.

### Network-based identification of new pharmacogenomics biomarkers in cancer

**Fig 5:**
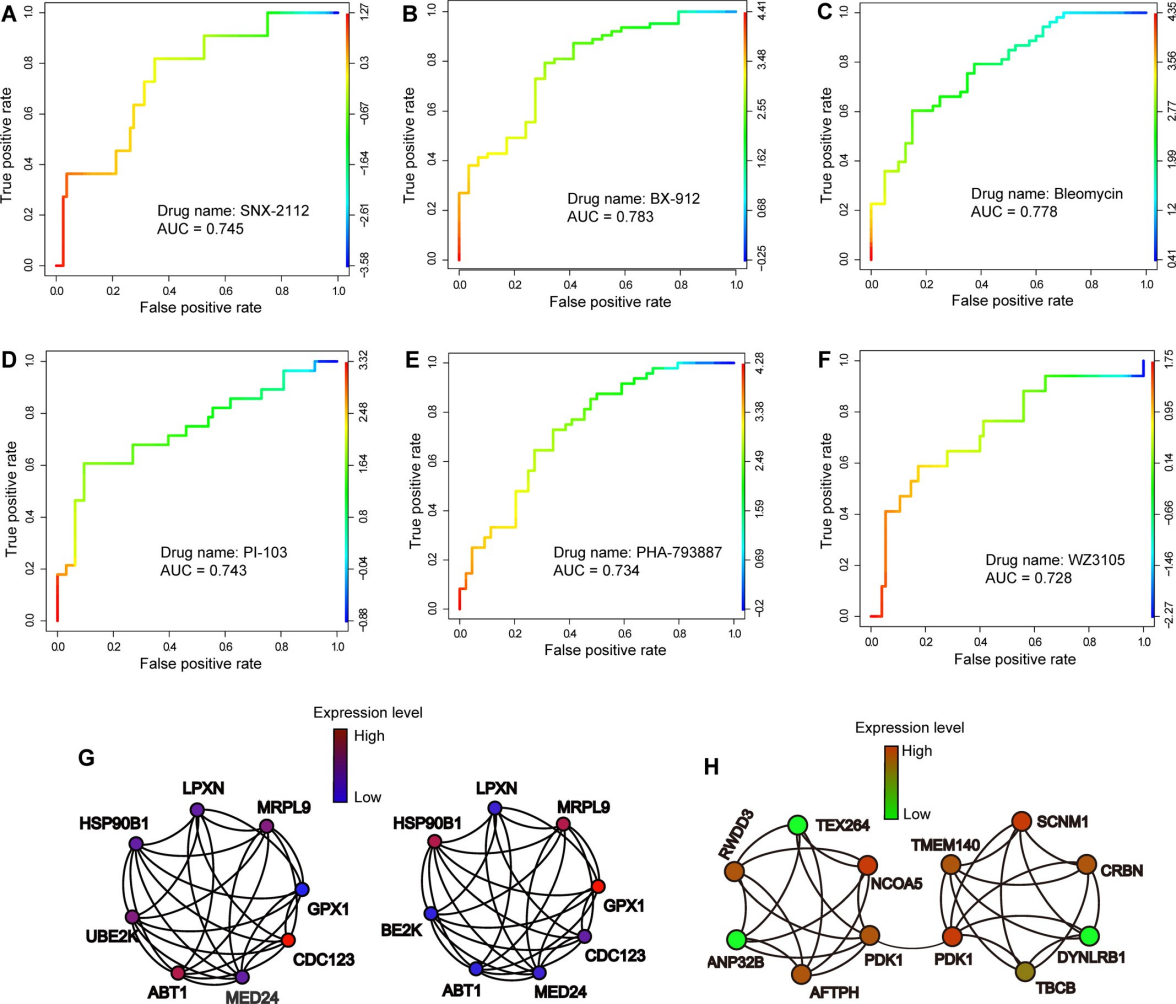
Cancer pharmacogenomics validation for COAC-predicted gene subnetworks. (**A** to **F**) The receiver operating characteristic (ROC) curves for six selected drugs: SNX-2112 (a selective Hsp90 inhibitor), BX-912 (a PDK1 inhibitor), Bleomycin (induction of DNA strand breaks), PHA-793887 (a pan-CDK inhibitor), PI-103 (a PI3K and mTOR inhibitor), and WZ3105 (also named GSK-2126458 or Omipalisib, a PI3K inhibitor). Drug IC_50_ values were predicted based on SVM regression models built by utilizing the COAC-inferred gene subnetworks as feature vectors (see Methods). The area under ROC curves (AUC) during 10-fold cross-validations were shown. In each ROC plot, the cutoff values at the corresponding curve positions are represented by the color keys. (**G** and **H**) Two COAC-inferred gene co-expression subnetworks for two selected drug targets on SNX-2112 (**G**) and BX-912 (**H**). The color key of each node indicates the weight of the genes in each subnetwork.

To examine the potential pharmacogenomics application of COAC, we collected robust multi-array (RMA) gene expression profiles and drug response data (IC_50_ [The half maximal inhibitory concentration]) across 1,065 cell lines from the Genomics of Drug Sensitivity in Cancer (GDSC) database [18]. We selected six drugs in this study based on two criteria: (i) the highest variances of IC_50_ among over 1,000 cell lines, and (ii) drug targets across diverse pathways: SNX-2112 (a selective Hsp90 inhibitor), BX-912 (a PDK1 inhibitor), Bleomycin (induction of DNA strand breaks), PHA-793887 (a pan-CDK inhibitor), PI-103 (a PI3K and mTOR inhibitor), and WZ3105 (also named GSK-2126458 and Omipalisib, a PI3K inhibitor). We first identified gene co-expression subnetworks from melanoma patients’ scRNA-seq data [11] by COAC. The COAC-inferred subnetworks with RMA gene expression profiles of bulk cancer cell lines were then transformed to a matrix: each column of this matrix represents a feature vector and each row represents a cancer cell line from the GDSC database [18]. We then trained an SVM regression model using the LIBSVM [19] R package with default parameters and linear kernel (see Methods). We defined cell lines whose IC_50_ were higher than 10 μM as drug-resistant cell lines (or non-antitumor effects), and the rest as drug sensitive cell lines (or potential antitumor effects). As shown in **Fig 5A–5F**, the area under the receiver operating characteristic curves (AUC) ranges from 0.728 to 0.783 across 6 drugs during 10-fold cross-validation, revealing high accuracy for prediction of drug responses by COAC-inferred gene subnetworks.

To illustrate the underlying drug resistance mechanisms, we showed two subnetworks identified by COAC for SNX-2112 (**Fig 5G**) and BX-912 (**Fig 5H**) respectively. SNX-2112, a selective Hsp90 (encoded by *HSP90B1*) inhibitors, has been reported to have potential antitumor effects in preclinical studies, including melanoma [20, 21]. We found that several *HSP90B1* co-expressed genes (such as *CDC123*, *LPXN*, and *GPX1*) in scRNA-seq data may be involved in SNX-2112’s resistance pathways (**Fig 5G**). *GPX1* [22] and *LPXN* [23] have been reported to play crucial roles in multiple cancer types, including melanoma. BX-912, a PDK1 inhibitor, has been shown to suppress tumor growth *in vitro* and *in vivo* [24]. **Fig 5H** shows that several PDK1 co-expressed genes (such as *TEX264*, *NCOA5*, *ANP32B*, and *RWDD3*) may mediate the underlying mechanisms of BX-912’s responses in cancer cells. *NCOA5* [25] and *ANP32B* [26] were reported previously in various cancer types. Collectively, COAC-inferred gene co-expression subnetworks from individual patients’ scRNA-seq data offer the potential underlying mechanisms and new biomarkers for assessment of drug responses in cancer cells.

## Discussion

In this study, we proposed a network-based approach to infer gene-gene relationships from large-scale scRNA-seq data. Specifically, COAC identified novel gene-gene co-expression in individual certain components (the subset of whole components) representing multiple cell types and cell phases, which can overcome a high degree of cell-cell variabilities from scRNA-seq data. We found that COAC reduced batch effects (**Fig 2**) and identified specific cell types with high accuracy (83%, **Fig 3C**) in two large-scale human scRNA-seq datasets. More importantly, we showed that gene co-expression subnetworks identified by COAC from scRNA-seq data were highly corrected with patients’ survival rate from TCGA data and drug responses in cancer cell lines. In summary, COAC offers a powerful computational tool for identification of gene-gene regulatory networks from scRNA-seq data, suggesting potential applications for the development of precision medicine.

There are several improvements in COAC compared to traditional gene co-expression network analysis approaches from RNA-seq data of bulk populations. Gene co-expression subnetwork identification by COAC is nearly unsupervised, and only a few parameters need to be determined. Since gene overlap among co-expression subnetworks is allowed, the number of co-expression subnetworks has a higher order of magnitude than the number of genes. Gene co-expression subnetworks identified by COAC can capture the underlying information of cell states or cell types. In addition, gene subnetworks identified by COAC shed light on underlying disease pathways (**Fig 4**) and offer potential pharmacogenomics biomarkers with well-defined molecular mechanisms (**Fig 5**).

We acknowledged several potential limitations in the current study. First, the number of predicted gene co-expression subnetworks is huge. It remains a daunting task to select a few biologically relevant subnetworks from a large number of COAC-predicted gene subnetworks. Second, as COAC is a gene co-expression network analysis approach, subnetworks identified by COAC are not entirely independent. Thus, the features used for computing similarities among cells are not strictly orthogonal. In the future, we may improve the accuracy of COAC by integrating the human protein-protein interactome networks and additional, already known, gene-gene networks, such as pathway information [27–29]. In addition, we could improve COAC further by applying deep learning approaches [30] for large-scale scRNA-seq data analysis.

In summary, we reported a novel network-based tool, COAC, for gene-gene network identification from large-scale scRNA-seq data. COAC identifies accurately the cell types and offers potential diagnostic and pharmacogenomic biomarkers in cancer. If broadly applied, COAC would offer a powerful tool for identifying gene-gene regulatory networks from scRNA-seq data in immunology and human diseases in the development of precision medicine.

## Methods and materials

### Pipeline of COAC

In COAC, a subnetwork is represented by the eigenvectors of its adjacency correlation matrix. In practice, the gene regulatory relationships represented by each subnetwork are not always unique. Those that occur in each subnetwork represent a superposition of two or several regulatory relationships, where each has a weight in gene subnetworks shown in **S3A Fig**. We thereby used multi-components (i.e., top eigenvectors with large eigenvalues) to represent the co-expression subnetworks. As shown in **S3B Fig**, a regulatory relationship between two genes can be captured in different co-expression subnetworks. Herein, we integrated matrix factorization [31] into the workflow of closed frequent pattern mining [32]. Specifically, the set of closed frequent patterns contains the complete itemset information regarding these corresponding frequent patterns [32]. Here, closed frequent pattern is defined that if two item sets appear in the same samples, only the super one is kept.

For a general gene expression matrix, to obtain a sparse distribution of genes in each latent variable, a matrix factorization method such as sparse principal component analysis (PCA) [33] can be chosen. In this study, because the scRNA-seq data matrix is highly sparse, singular value decomposition (SVD) is chosen for matrix factorization (i.e., the SVD of A is given by UσV*). The robust rank r is defined in the **S1 Text**. Components that are greater than rank r are selected and then each attribute is treated as the linearly weighted sum of components (D_i_ = w_i1_ **P**_1_ + w_i2_ **P**_2_ + w_i3_ **P**_3_ …w_ir_ **P**_r_). The projection of gene distribution *i* over principal component *j* can be expressed as $\frac{{D_{i}}^{t}P_{j}}{\left\|D_{i}\|\|P_{j}\right\|}$, where ‖**P**_*j*_‖ = 1. Then, $D(i,j)=\frac{{D_{i}}^{t}P_{j}}{\left\|D_{i}\|\|P_{j}\right\|}=\frac{{D_{i}}^{t}P_{j}}{\left\|D_{i}\right\|}=\frac{w_{ij}}{\left\|D_{i}\right\|}$ and $-1<\frac{{D_{i}}^{t}P_{j}}{\left\|D_{i}\right\|}<1$. The projection of each attribute distribution over each principal component distribution is illustrated in **S4A Fig**. In practice, single cell data are always sparse. For component *j*, most elements in the collection of D(i,j)|j are zero. Several thresholds are determined by F-distribution. For a component *j*, the mean and the variance of collection D(i,j)|j is m and s^2^. Then the F-distribution with degree of freedom 1, and degree of freedom N-1 (N is the number of attributes) is:

$$F_{\left(1,N-1\right)}(x)=\frac{{\left(x-m\right)}^{2}}{s^{2}}$$ (1)

The P-value for a element x in collection D(i,j)|j is the extreme upper tail probability of this F-distribution. The threshold of the collection D(i,j)|j is divided into two groups. In one group, the P-value of all element should be below a pre-defined threshold. The detailed process for obtaining the thresholds is described in the **S1 Text**. Herein, the cutoff of P-value for F-distribution ranges from 0.01 to 0.05. Subsequently, we defined the mapping rule using these thresholds.

$$\left\{ \begin{array}{c} 1\;\mathrm{if}\;{\mathrm{threshold}\;P}_{j}<\frac{D_{x}{}^{t}P_{j}}{\left\|D_{x}\right\|}<1\;(\mathrm{Gain})\\ 0\;\mathrm{if}\;\mathrm{threshold}\;N_{j}<\frac{D_{x}{}^{t}P_{j}}{\left\|D_{x}\right\|}<\mathrm{threshold}\;P_{j}\;(\mathrm{Non}-\mathrm{effect})\\ -1\;\mathrm{if}-1<\frac{D_{x}{}^{t}P_{j}}{\left\|D_{x}\right\|}<\mathrm{threshold}\;N_{j}\;(\mathrm{Loss}) \end{array}\right.$$ (2)

The pipeline is shown in **S4B** and **S4C Fig**. In the (1/0) sparse matrix, each row represents a component while each column represents an attribute (gene). The association rule is consisted of: (i) one is an attribute (gene) collection and (ii) the other is a component collection. The position in the binary distribution matrix of any pair with the Cartesian product of the two collections is always 1. This position is shown in **S4D** and **S4E Fig**.

For each association rule, the attribute collection should have maximal component collection. For example, for association rules {X Y Z} {M}, {X Y} {M}, {X Y} {M N}, only the maximal {X Y} {M N} is allowed. And the closed association rule states that if two rules have the same component collections, only the maximal attribute collection is preserved and kept. For association rules {X Y Z} {M N}, {X Y} {M N}, {Y Z} {M N}, and {X Z} {M N}, with the same component collection {M, N}, only the maximal {X Y Z} {M N} is kept, whereas the others are removed. The process of efficient enumeration of all significant association rules (gene subnetwork) is described in the **S1 Text**. The subnetwork and gene distribution of selected components are obtained directly by applying the association rule, and the gene subnetwork is treated as the largest connected component (graph) from co-expression networks of scRNA-seq profiles. Finally, two metrics are introduced for filtering. The *average correlation* among genes in each subnetwork is a measure of the homogeneity of genes with selected components. The *average component ratio* denotes the average of how much of the whole component space is occupied by the selected components.

$$\mathrm{Average}\;\mathrm{Correlation}=\left(\frac{1}{n(n-1)}\right)\sum_{i,j\in \{X,Y,Z\},j,i\neq j}\mathrm{Correlation}\left(A_{i},A_{j}\right)|M,N$$ (3)

$$\mathrm{Component}\;\mathrm{Ratio}\;\mathrm{of}\;A_{i}=\frac{\|A_{i}\|2|selected\;components}{\|A_{i}\|2}$$ (4)

$\mathrm{Average}\;\mathrm{Component}\;\mathrm{Ratio}=\frac{1}{N}\sum Component\;Ratio\;of\;A_{i}$(A_i_ ∈ attribute collection of a closed associate rule) (5)

The processes of obtaining the average correlation and the average component ratio are provided in the **S1 Text**.

The final largest connected component subnetwork is represented by several eigenvectors with large eigenvalues, which are calculated from the correlation matrix. These eigenvectors are used to map each record of the gene expression profile into individual numerical values (feature vectors).

$$\mathrm{Feature}\;\mathrm{vector}=S^{\;}F^{t}/\|S{\|}_{2}\left(\|F{\|}_{2}=1\right)$$ (6)

Where **S** is the gene expression vector for each cell, and **F** is the first eigenvector of the component matrix. If several principal components exist, then the feature value becomes the sum of components multiplied by the attenuation coefficient.

$$\mathrm{Feature}\;\mathrm{vector}=S\;F_{1}^{t}/\|S{\|}_{2}+\left(\sigma_{2}/\sigma_{1}\right)S\;F_{2}^{t}/\|S{\|}_{2}+\left(\sigma_{3}/\sigma_{1}\right)S\;F_{3}^{t}/\|S{\|}_{2}\ldots (\|F_{1}^{t}{\|}_{2}=1,\|F_{2}^{t}{\|}_{2}=1\ldots )$$ (7)

Where σ_1_, σ_2_, σ_3_,…,σ_*v*_ are the eigenvalues of the gene clustering (subnetwork) correlation matrix, and $F_{1}^{t},F_{2,}^{t}\ldots$ are the eigenvectors of gene clustering correlation matrix.

### Cell type alignment by COAC

The purpose of cell type alignment was to label cell types of each cell under different conditions. Cell types with the same labels under each condition were then clustered. Subsequently, differential expression analyses were performed for various conditions of each cell type. Finally, surrogate variable analyses [34] were performed to remove the batch effects. We used the limma [35] method (**S5B Fig**) for the differential expression analysis of the differently conditioned cell types.

The scRNA-seq data (GEO accession ID: GSE96583) that was used to test the batch effect elimination was collected from PBMC peripheral blood mononuclear cells of SLE patients [7,8]. In total, 14,032 cells with 13 aligned PBMC subpopulations under resting and interferon β (IFN-β)-stimulated conditions were collected [8]. In addition, we also collected 29,067 cells from two controls as the control group [7]. For the training dataset, the variances of the feature vectors (COAC-identified subnetworks) between the case group and the control group were calculated and was regarded as differential variances. The variances of the feature vectors of the merged group of the case group and the control group were regarded as background variances. For each feature, the ratio of the differential variance and background variance was defined as F-score, which measured how much this feature can distinguish cells in a case group *versus* a control group. The F-score distribution for 93,951 features is described in **S6 Fig**. Using a critical point of 2.4 as a threshold (**S6 Fig**), 8,331 features with F-score higher than the threshold were kept. For comparison, we used 2,657 genes which were used as biomarkers previously as the feature vector [8].

### Cell type identification by COAC

The scRNA-seq data of mouse kidney with well-annotated cell types were collected from a previous study [10]. By stringent quality controls described previously [10], a total of 43,745 cells selected from the original 57,979 cells were used in this study. The entire dataset was randomly divided into the training set (21,873 cells) and the test set (21,872 cells). The detail of prediction model construction can be found in cell type alignment pipeline (**S5 Fig**). For the validation part, cell type was predicted using the training model. For each cell, the scores for cell types were calculated. Then all cells were plotted by t-SNE algorithm [9]. The results of cell type prediction were displayed in the confusion matrix.

### Identification of new prognostic biomarkers by COAC

We collected the melanoma patients’ scRNA-seq data with well-annotated cell types from a previous study [11]. The bulk RNA-seq data and clinical profiles for melanoma patients were collected from the TCGA website [13]. The gene expression values in the scRNA-seq dataset were transformed as log (TPM_ij_+1), where TPM_ij_ refers to transcript-per-million (TPM) of gene *i* in cell *j*. The gene expression value in the bulk RNA-seq dataset was transformed in the same way.

The sub-network list was obtained from melanoma scRNA-seq dataset [11] by COAC. Sub-networks then were transformed to feature vectors. Two top sub-networks with the highest co-expressed correlation in melanoma cell type and one top sub-network with the highest co-expressed correlation in T cells were evaluated. The co-expression values were calculated with RNA-seq gene expression of melanoma patients from TCGA [13]. Survival analysis was conducted using an R survival package [36].

### Identification of new pharmacogenomics biomarkers by COAC

We downloaded drug response data (defined by IC_50_ value) and gene bulk expression profiles in cancer cell lines from the GDSC database [18]. The component co-expression sub-networks were identified from the melanoma patients’ scRNA-seq data with well-annotated cell types from a previous study [11]. For scRNA-seq data, genes that had a ratio of expressed cells less than 0.03 were removed. Herein, we kept the top 0.1~0.01 percent subnetworks with the highest correlation as feature vectors. We predicted each drug’ IC_50_ value by LIBSVM [19] R package with default parameters and linear kernel. The ROC curves for the result of drug response were plotted using the R package.

## Supporting information

S1 Text Supplemental methods. (PDF)

S1 Fig Distribution of feature selection between malignant cells and control cells from scRNA-seq data of individual melanoma patients. (PDF)

S2 Fig Survival analysis for top 12 selected significant COAC-inferred gene co-expression subnetworks from scRNA-seq data in Melanoma. (PDF)

S3 Fig A diagram illustrating the process of gene co-expression subnetwork identification by COAC. (PDF)

S4 Fig A diagram illustrating matrix factorization method for gene co-expression subnetwork identification. (PDF)

S5 Fig A diagram illustrating of the pipeline of cell type identification by COAC. (PDF)

S6 Fig Distribution of the ratio (F-score) of the differential variance and background variance. (PDF)

S7 Fig A diagram illustrating the processes of binary distribution matrix analysis and principal components contribution analysis. (PDF)
