# Supplementary material for: A component overlapping attribute clustering (COAC) algorithm for single-cell RNA sequencing data analysis and potential pathobiological implications
Source: PLoS Comput Biol. 2019 Feb 19;15(2):e1006772. doi: 10.1371/journal.pcbi.1006772 (PMC6396937; doi:10.1371/journal.pcbi.1006772)
Supplement: S1 Text — (PDF) [file pcbi.1006772.s001.pdf]

Supporting Information

Title: A Component Overlapping Attribute Clustering (COAC) algorithm for Single-cell RNA Sequencing Data Analysis and Potential Pathobiological Implications

To whom correspondence should be addressed:

Feixiong Cheng, Ph.D.

Genomic Medicine Institute

Lerner Research Institute, Cleveland Clinic

9500 Euclid Avenue, Cleveland, Ohio 44195

Email: chengf@ccf.org

Phone: +1-216-4447654; Fax: +1-216-6361609

Supplemental Methods

The definition of Robust Rank

D_i =

	Attribute i
Sample 1	0.23
Sample 2	0.67
Sample 3	0.78

σ_k(**A**) denotes the *i*th largest singular value of **A**. Define the minimal r as the robust rank of **A** that satisfies the constraint:

$$(\sigma_1(\mathbf{A})^2 + \sigma_2(\mathbf{A})^2, \ldots\ldots, + \sigma_r(\mathbf{A})^2)/(\sigma_1(\mathbf{A})^2 + \sigma_2(\mathbf{A})^2, \ldots\ldots, + \sigma_r(\mathbf{A})^2 + \ldots\ldots + \sigma_n(\mathbf{A})^2) > \theta \quad (1)$$

θ is the tolerant threshold was defined by the user, which is always greater than zero.

Selection of the threshold

An F-distribution for the collection of $\mathrm{D(i,j)|j}$ for component *j* can be obtained.

The mean of a collection of $\mathrm{D(i,j)|j}$ is $\mathrm{m} = \frac{\boldsymbol{1}}{\boldsymbol{N}}\sum_{\boldsymbol{i=1}}^{\boldsymbol{N}} \mathrm{D(i,j)}\ |\mathrm{j}$. (2)

The square of a collection of $\mathrm{D(i,j)|j}$ is $\mathrm{s}^2 = \frac{\boldsymbol{1}}{\boldsymbol{N-1}}\sum_{\boldsymbol{i=1}}^{\boldsymbol{N}}(\mathrm{D(i,j)} - \mathrm{m})^2\ |\mathrm{j}$. (3)

Then the F-test with degrees of freedom 1, and degrees of freedom N-1 (N is the number of attributes) is:

$$\mathrm{F}_{(1,\mathrm{N}-1)}(\mathrm{x}) = \frac{(\mathrm{x}-\mathrm{m})^2}{\mathrm{s}^2} \quad (4)$$

Extreme upper tail probability is computed for an element in a collection of $\mathrm{D(i,j)|j}$.

$P(x > D(i,j)) < CUTOFF$. The $CUTOFF$ of P-value is user defined.

We divided the collection of $D(i,j)|j$ into two groups for each component, and for one group, the P-value is always less than user defined cutoff.

In summary, the final optimal threshold is obtained by iteratively splitting, in each iterative splitting, the threshold was determined and the upper tail probability for this threshold was calculated. If this p-value is small enough (less than a cutoff) then end this iteration. This divide strategy is not strictly sensitive to the defined P-value cutoff. So that a small P-value cutoff variable won't change the result too much. In gene decomposition, we treated the negative and positive elements of $D(i,j)|j$ separately. For each component, we can obtain two thresholds in this way. The detail of divide strategy was provided in a pseudocode way as below:

1. For each component j (positive part and negative part) :
2. Sort the atrributes according the atrribute scores in this component *j* ($\{D(i,j)\}|j$).
3. For each attribute *i*, the attributes group was split into two part. In one part the attribute score is always below D(i,j), while it is opposite for another group.
4. The ratio of variance between groups and population variance was caculated.
5. The attribute which has the highest ratio was chosen as a threshold. The collection of attribute was splited by this threshold with order of atrribute scores for component *j*. If P-value of all elements is less than user-defined cutoff, this iteration will be terminated and only the attributes which belong to this group are kept and move to step 2 for the next iteration.

Closed association rules enumeration

We used a node to represent a component. Each attribute is represented by a unique attribute ID, and an attribute ID list is assigned to each node. We constructed a prefix tree using this kind of nodes. In this prefix tree, the path from a node to the root node represents a component collection. And the attribute ID list of this node is the intersection of attribute ID lists for all nodes in this path. We constructed an attribute ID list library to store the attribute ID list of the nodes that have been searched.

The rules of how to traveling the prefix tree are defined as below:

The node IDs in the prefix tree are ordered. The father node ID must be greater than children node ID, and for children nodes from same father node, the left children nodes must be greater than right children nodes. More strictly this node ID is one larger than its left sibling node ID. If a node has no left sibling node, then it must be larger than the father node. And the root node is the largest node ID. Any combination of node IDs can be obtained by traveling this prefix tree in this way. The combination of node IDs is represented by the ID path from a node to the root node.

Proposition 1

In depth-first traveling this prefix tree, if combination A of node IDs is a part of combination B of node IDs, then combination B must be traveled firstly.

Proof

Case 1:

The path of node IDs combination A is < p_1, p_2, …, p_k >. The path of node IDs combination B is < b_1, …, b_k, p_1, …, b_m, …, p_k…, b_n >. As the node ID b_1 is greater than node ID p_1, the prefix < b_1 is firstly traveled before the prefix < p_1.

Case 2:

The path of node IDs combination A is < p_1, p_2, …, p_m, p_n, …, p_k >. The path of node IDs combination B is < p_1, p_2, …, p_m, b_n, …, p_n, …, b_n >. < p_1, p_2, …, p_m> is the maximum common prefix string of two node ID combinations. Because the node ID b_n is greater than node ID p_n, the prefix < p_1, p_2, …, p_m >, b_n is traveled before the prefix < p_1, p_2, …, p_m, p_n >.

Proposition 2

In a depth-first traveling prefix tree, if a node's the attribute ID list is not the same as any children nodes, this node is closed (maximum node IDs with this attribute ID list).

Proof

According to **Proposition 1**, if this node is not maximum node IDs with this attribute ID list, there is at least a children node's attribute-ID list is the same as this node.

Proposition 3

If there are two node IDs combinations A and B. One is a part of another (A>B) and they have the same attribute-ID list. For any offspring node a_i of node A, there is an offspring

node, b_j, of node B, satisfying that b_j is a part of a_i, and they have the same attribute-ID list.

Proof

If < i_{b1}, i_{b2}, …, i_{bn}> is the node ID path sequence for node B, then any offspring node of node B can be indicated by < i_{b1}, i_{b2}, …, i_{bn}, p_{bn+1}, p_{bn+2}, …, p_{bm} >. If < i_{a1}, i_{a2}, …, i_{an}> is the node ID path sequence for node A, we will add the postfix ID sequence of b_j to ID sequence of node A to construct an offspring node a_i of node A. the ID sequence of a_i is < i_{a1}, i_{a2}, …, i_{an} , p_{bn+1}, p_{bn+2}, …, p_{bm} >. Because the node ID path sequence of B is a part of the node ID path sequence of A, and they have the same attribute-ID list and a_i and b_j have the same postfix ID sequence so b_j is a part of a_i and they have the same attribute-ID list.

The procedure for enumerating all closed association rules (attribute collection and component collection) were described as below:

1. For each node **n** in a prefix tree with the depth-first searching:

   If the length of attribute-list for node **n** is less than a support threshold, then remove this node and its offspring nodes.

   If the attribute-list of this node **n** would be found in the attribute-list library, then remove this node and its offspring nodes (According to **Proposition 3**).

2. Check the length of attribute-list for all children nodes of this node **n**:

If there are not any children node's attribute-list has the same length with parent node **n**, then report the node ID path of node **n** and the attribute list of this node, the attribute-list of this node is recorded in the attribute-list library as well (According to **Proposition 2**).

Average correlation and the average component ratio

The 2-norm of attribute X can be expanded by a series of orthogonal normalize components.

$$A_i = w_{1i}\mathbf{P}_1 + w_{2i}\mathbf{P}_2 + \dots w_{ri}\mathbf{P}_r \dots \quad (5)$$

$$\| A_i \|_2 = \sqrt[2]{w_{1i}^2 + w_{2i}^2 + \dots w_{ri}^2 \dots} \quad (6)$$

The truncated 2-norm was defined as 2-norm calculated with specific components.

Such as $\| A_i \|_2 = \sqrt[2]{w_{ni}^2 + w_{mi}^2 + w_{li}^2}$ denote as $\| A_i \|_2 \mid n,m,l$ (7)

It means that 2-norm calculated only consider components: $\mathbf{P}_n, \mathbf{P}_m, \mathbf{P}_l$. In the same way, the truncated correlation was defined as correlation calculated with specific components.

Such as $\mathrm{cor}(A_i, A_j) = \frac{w_{nj}w_{ni} + w_{mj}w_{mi} + w_{lj}w_{li}}{\sqrt[2]{w_{ni}^2 + w_{mi}^2 + w_{li}^2}\ \sqrt[2]{w_{nj}^2 + w_{mj}^2 + w_{lj}^2}}$ denote as $\mathrm{cor}(A_i, A_j) | n, m, l$ (8)

It means that the correlation between A_i, A_j calculated only consider components: $\mathbf{P}_n, \mathbf{P}_m$, $\mathbf{P}_l$. For a closed associate rule we calculated the average correlation among the attribute collection only considering components in the component collection.

For a closed associate rule {XYZ} {M N}

Average correlation = $(\frac{1}{n(n-1)}) \sum_{i,j \in \{X,Y,Z\}, i \neq j} \mathrm{Cor}(A_i, A_j) \mid M,N$ (9)

The average correlation for each closed associate rule was calculated as criteria for associate rule screening. In practice, we chose 0.99~0.98 as *average correlation* threshold. Only closed associate rules with high average correlation were kept.

The closed associate rule with high average correlation indicated a kind of attribute (gene) pattern, but it is still not sure whether this pattern is useful in practice. We thereby introduced the second measure to quantify how important of this pattern playing in whole components.

The selected components contain the components in the component collection in this closed associate rule and the additional components which don't have significant value in binary distribution matrix. As depicted in **S7 Fig**. For each attribute, we calculated the ratio of 2-normal of this attribute with selected components and 2-normal of this attribute with whole components. Denote as *Component Ratio*.

$$\textit{Component Ratio of } A_i = \frac{\| A_i \|_2 | \text{ selecteted components}}{\| A_i \|_2} \quad (10)$$

$$\textit{Average Component Ratio} = \frac{1}{N} \sum \text{Component Ratio of } A_i$$

$$(A_i \in \text{attribute collection of a closed associate rule}) \quad (11)$$

Supplemental Figures

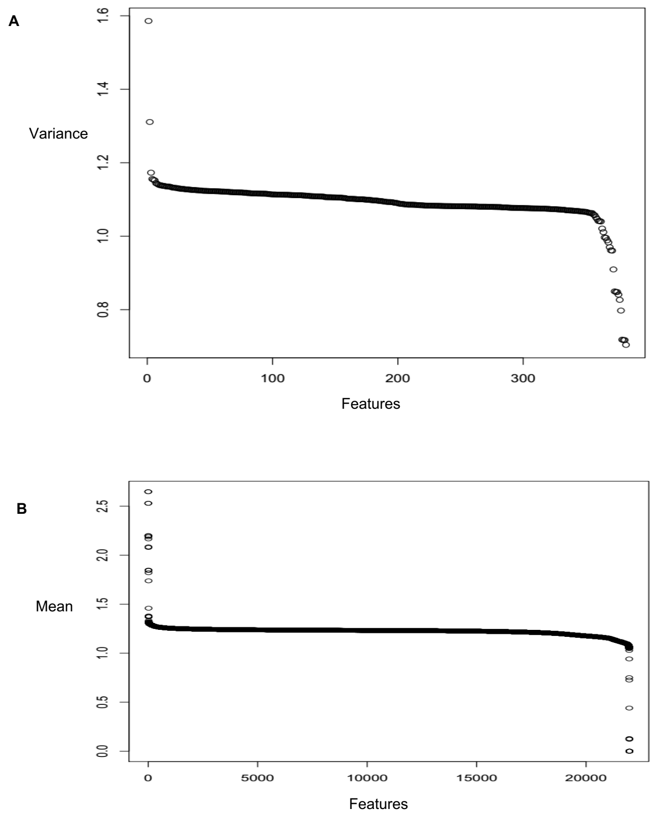

S1 Fig. Distribution of feature selection between malignant cells versus control cells from scRNA-seq data of individual melanoma patients. (**A**) Distribution of ratio of variances between malignant cells and control cells for selected features from scRNA-seq data of melanoma patients [1]. (**B**) Distribution of ratio of means between malignant cells and control cells for all features.

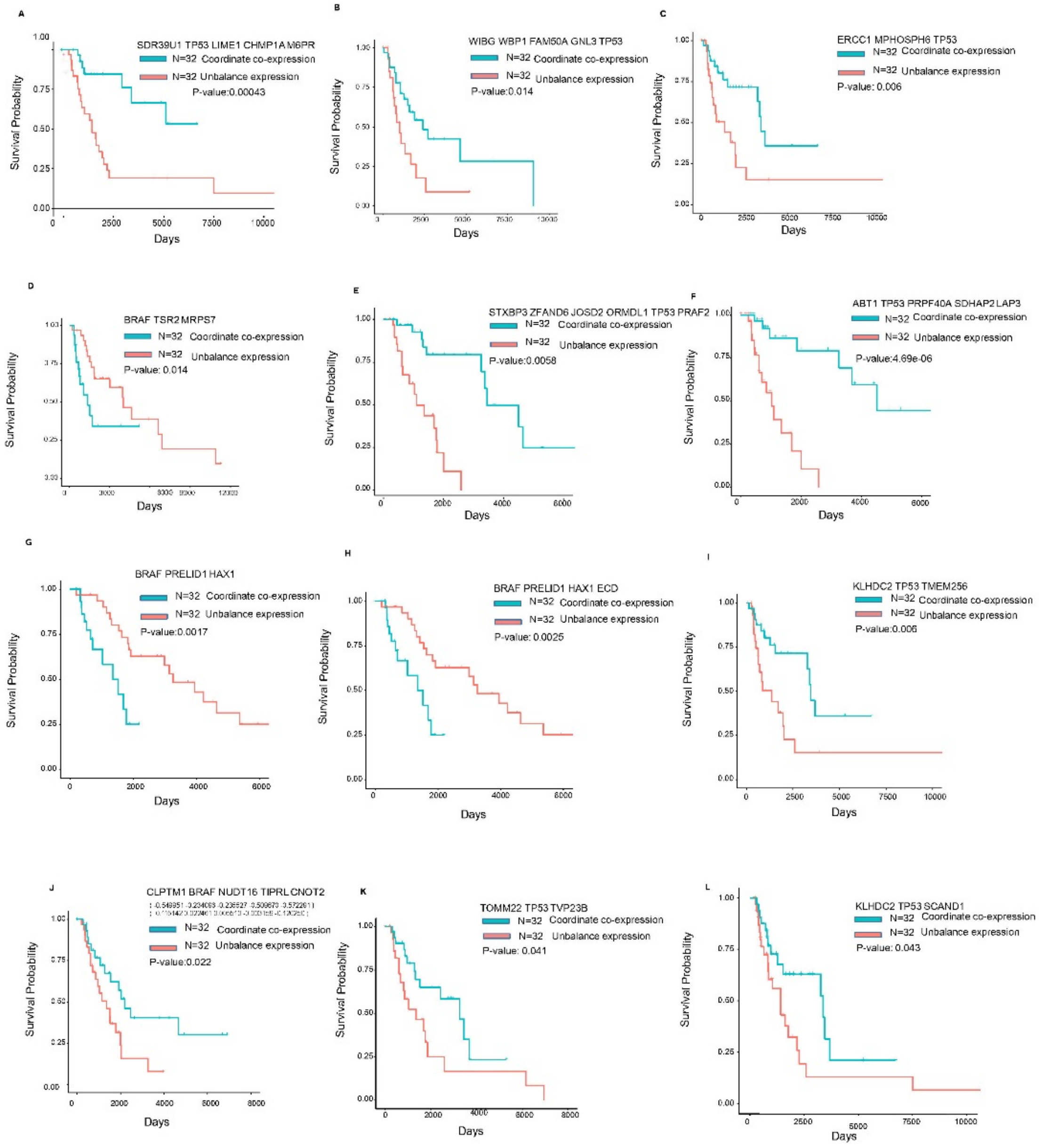

S2 Fig. Survival analysis for top 12 selected COAC-inferred gene co-expression subnetworks from scRNA-seq data in Melanoma patients. The top selected subnetwork for each survival analysis was highlighted in each subfigure. The bulk RNA-seq data and clinical profiles for each melanoma patients were collected from TCGA website [2]. Survival analysis was conducted for these two groups using the R survival package [3].

A

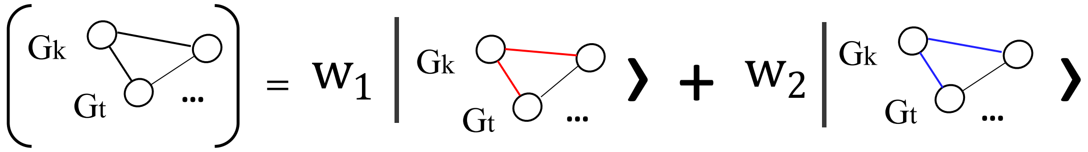

B

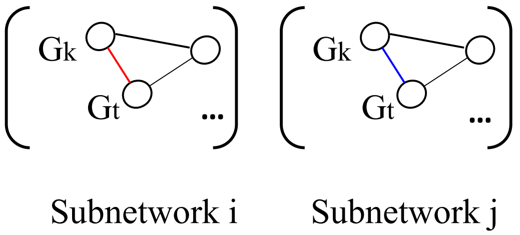

S3 Fig. A diagram illustrating the process of gene co-expression subnetwork identification by COAC. (**A**) Each co-expression sub-network can be treated as a superposition of two different gene expression state. (**B**) The gene co-expression relationship can be represented by different subnetworks.

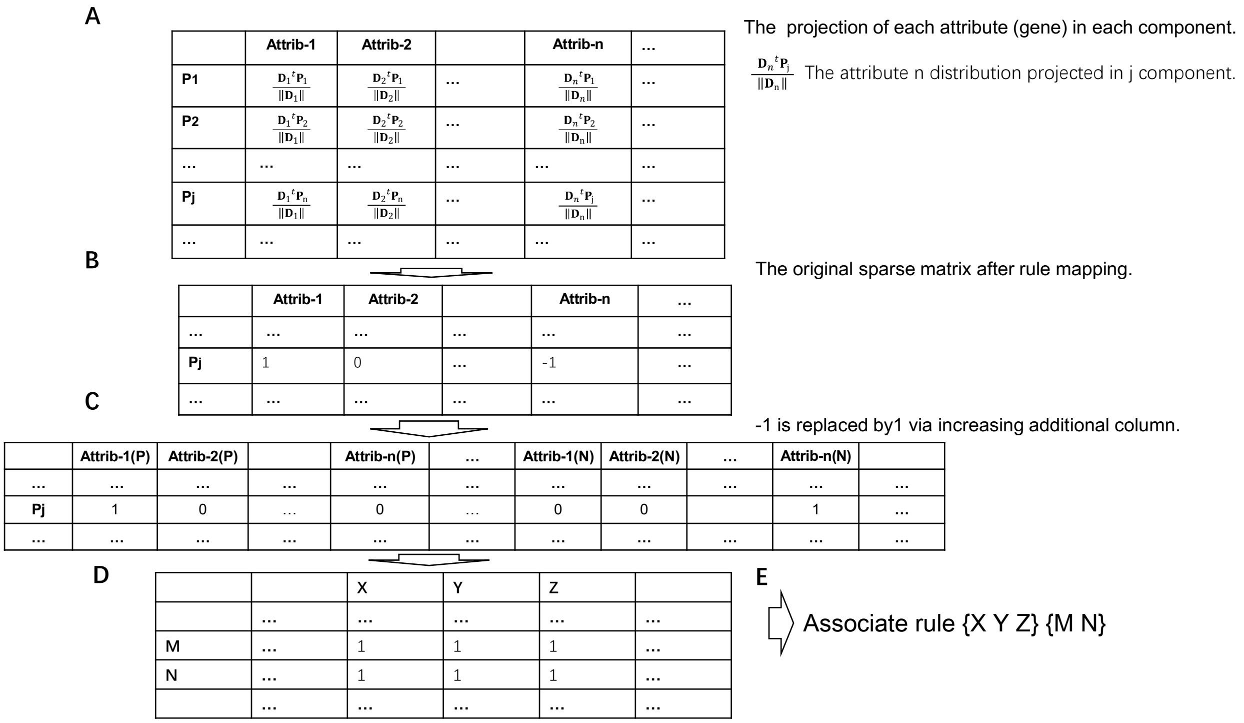

S4 Fig. A diagram illustrating matrix factorization method for gene co-expression subnetwork identification. (**A** and **B**) The projection of each attribute distribution over each principal component distribution. (**C**) A binary matrix composed of 1 or 0 after rule mapping. (**D** and **E**) Associate rules were obtained from the sub-tables of binary matrix whose all elements are no-zero. For the domain of “-1”, we translated “–1” to “1” by increasing an additional column.

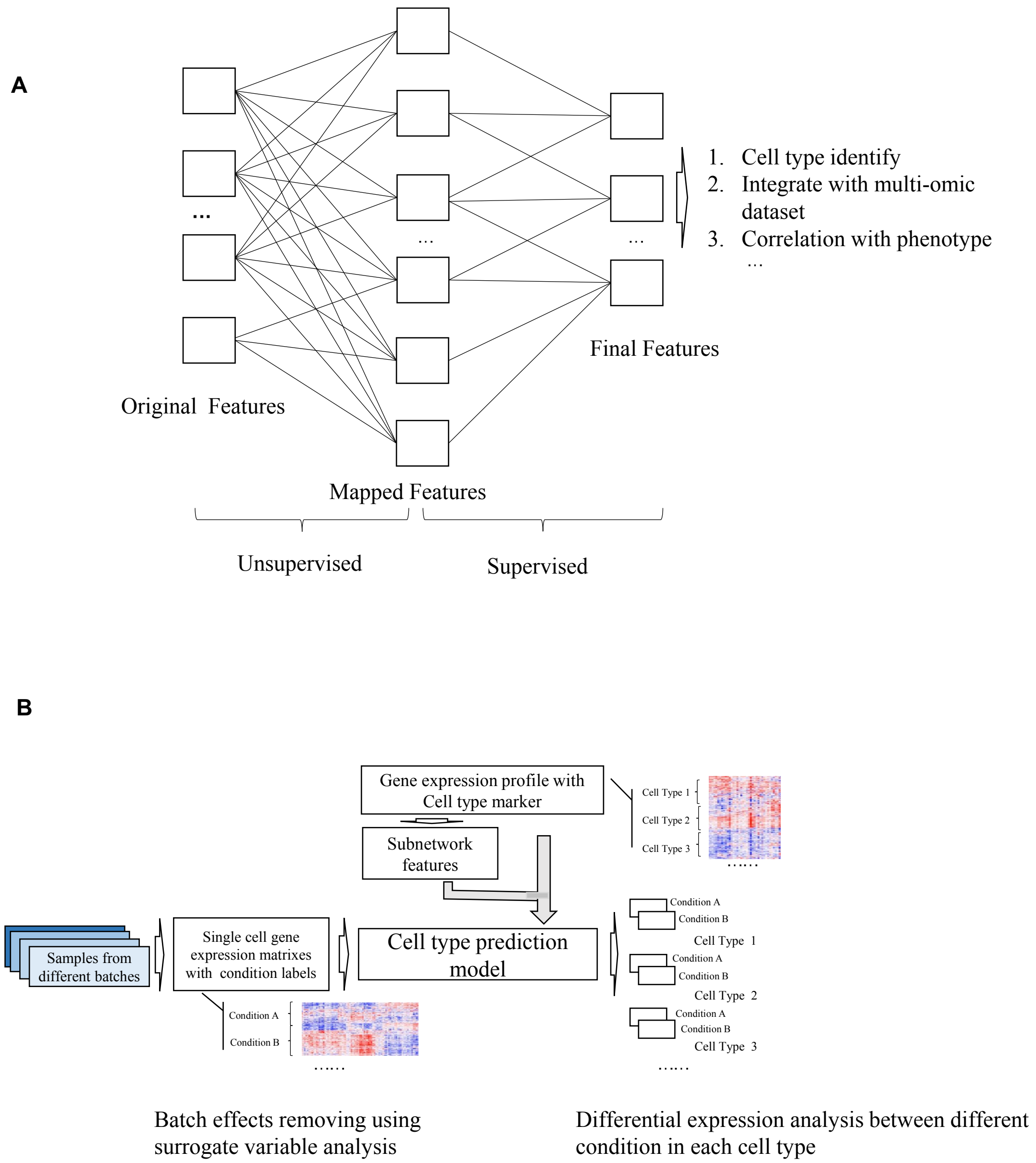

S5 Fig. A diagram illustrating of the pipeline of cell type identification by COAC. (**A**) A diagram shows the pipeline from single gene to gene co-expression network features and the final features will be obtained from gene co-expression subnetworks in a supervised way. (**B**) A pipeline illustrating for cell type identification.

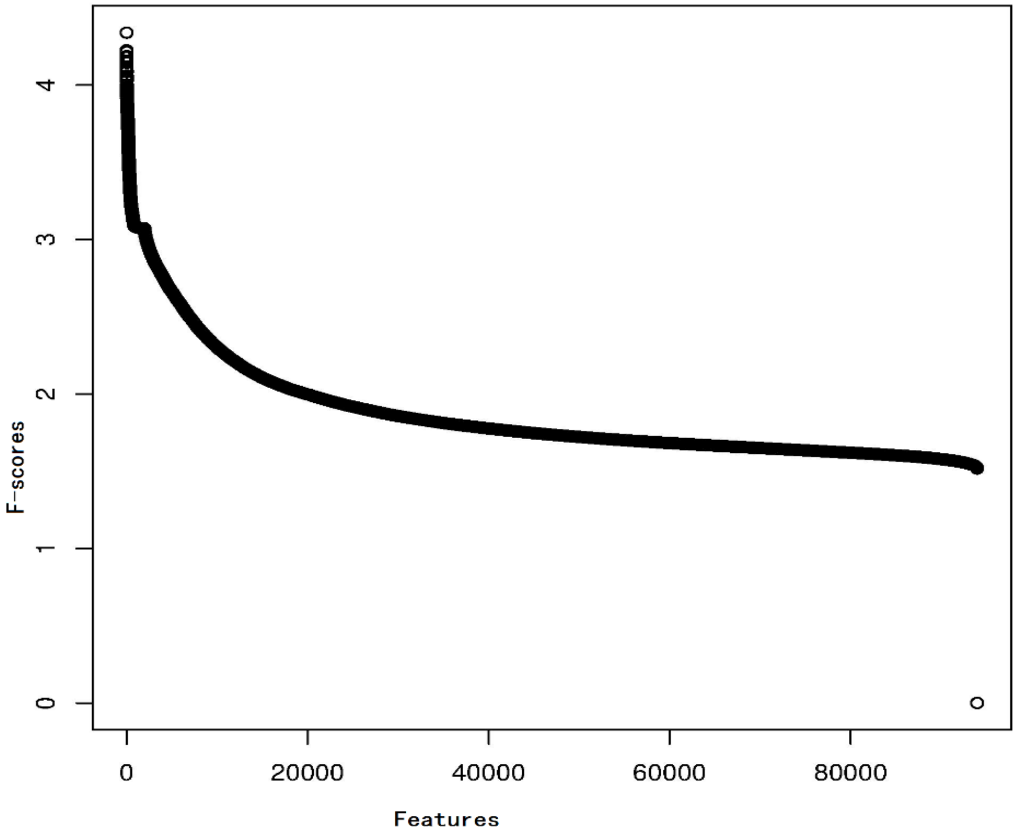

S6 Fig. Distribution of the ratio (F-score) of the differential variance and background variance.

Binary distribution matrix

Attribute Name / Component ID	RMND1	ANKRD54	MC2R
2015	1	1	1
641	1	1	1
281	X	X	X
609	X	X	X
899	X	X	X
1414	X	X	X
...	...	...	...

X means it is 1/0 , not always 1

Principle components contribution in each attributes

Attribute Name / Component ID	RMND1	ANKRD54	MC2R
2015	-4.012	-4.903	-1.069
641	4.436	3.949	0.872
281	1.093	1.369	0.273
609	1.261	1.342	0.286
899	-1.577	-1.688	-0.396
1414	-1.238	-1.115	-0.269
...	....	....	....

The original components in this closed associate rule which have significant value in binary distribution matrix. (components 2015, 641)

The components which have similar pattern with original components. (components 281, 609, 899, 1414, ...)

S7 Fig. A diagram illustrating the processes of binary distribution matrix analysis and principle components contribution analysis. The selected components containing all components whose distribution is similar with the components in the component collection of this closed associate rule.

References

1. Tirosh I, Izar B, Prakadan SM, Wadsworth MH, Treacy D, Trombetta JJ, et al. Dissecting the multicellular ecosystem of metastatic melanoma by single-cell RNA-seq. Science. 2016; 352(6282):189-96.
2. Bewick V, Cheek L, Ball J. Statistics review 12: survival analysis. Crit. Care. 2004; 8(5):389.
3. Therneau T, Lumley T. Survival: Survival analysis, including penalised likelihood. R package version 2.35-7. R foundation for Statistical Computing 2011.
