# Supplementary material for: A component overlapping attribute clustering (COAC) algorithm for single-cell RNA sequencing data analysis and potential pathobiological implications
Source: PLoS Comput Biol. 2019 Feb 19;15(2):e1006772. doi: 10.1371/journal.pcbi.1006772 (PMC6396937; doi:10.1371/journal.pcbi.1006772)
Supplement: S1 Fig — (PDF) [file pcbi.1006772.s002.pdf]

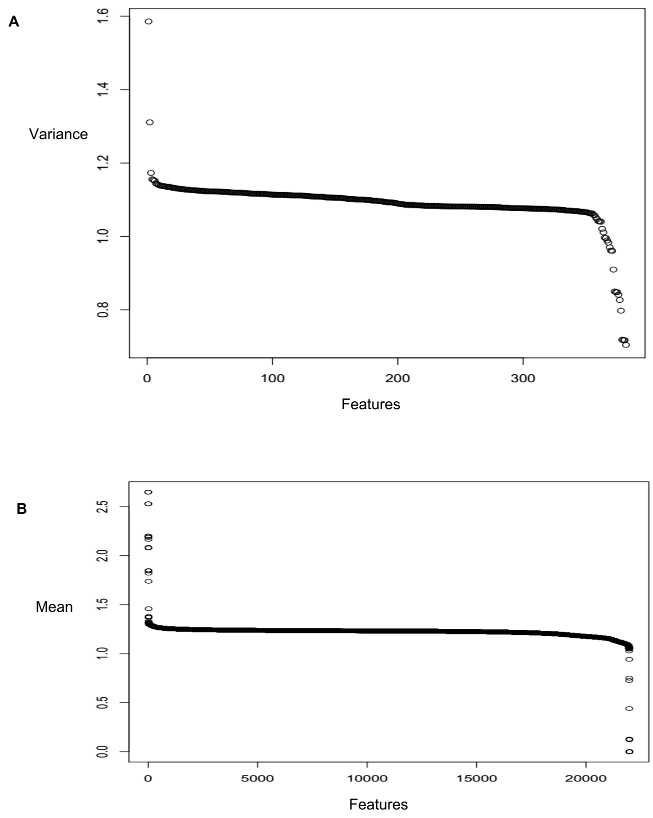

S1 Fig. Distribution of feature selection between malignant cells versus control cells from scRNA-seq data of individual melanoma patients. (**A**) Distribution of ratio of variances between malignant cells and control cells for selected features from scRNA-seq data of melanoma patients [1]. (**B**) Distribution of ratio of means between malignant cells and control cells for all features.
