# Supplementary material for: A component overlapping attribute clustering (COAC) algorithm for single-cell RNA sequencing data analysis and potential pathobiological implications
Source: PLoS Comput Biol. 2019 Feb 19;15(2):e1006772. doi: 10.1371/journal.pcbi.1006772 (PMC6396937; doi:10.1371/journal.pcbi.1006772)
Supplement: S2 Fig — (PDF) [file pcbi.1006772.s003.pdf]

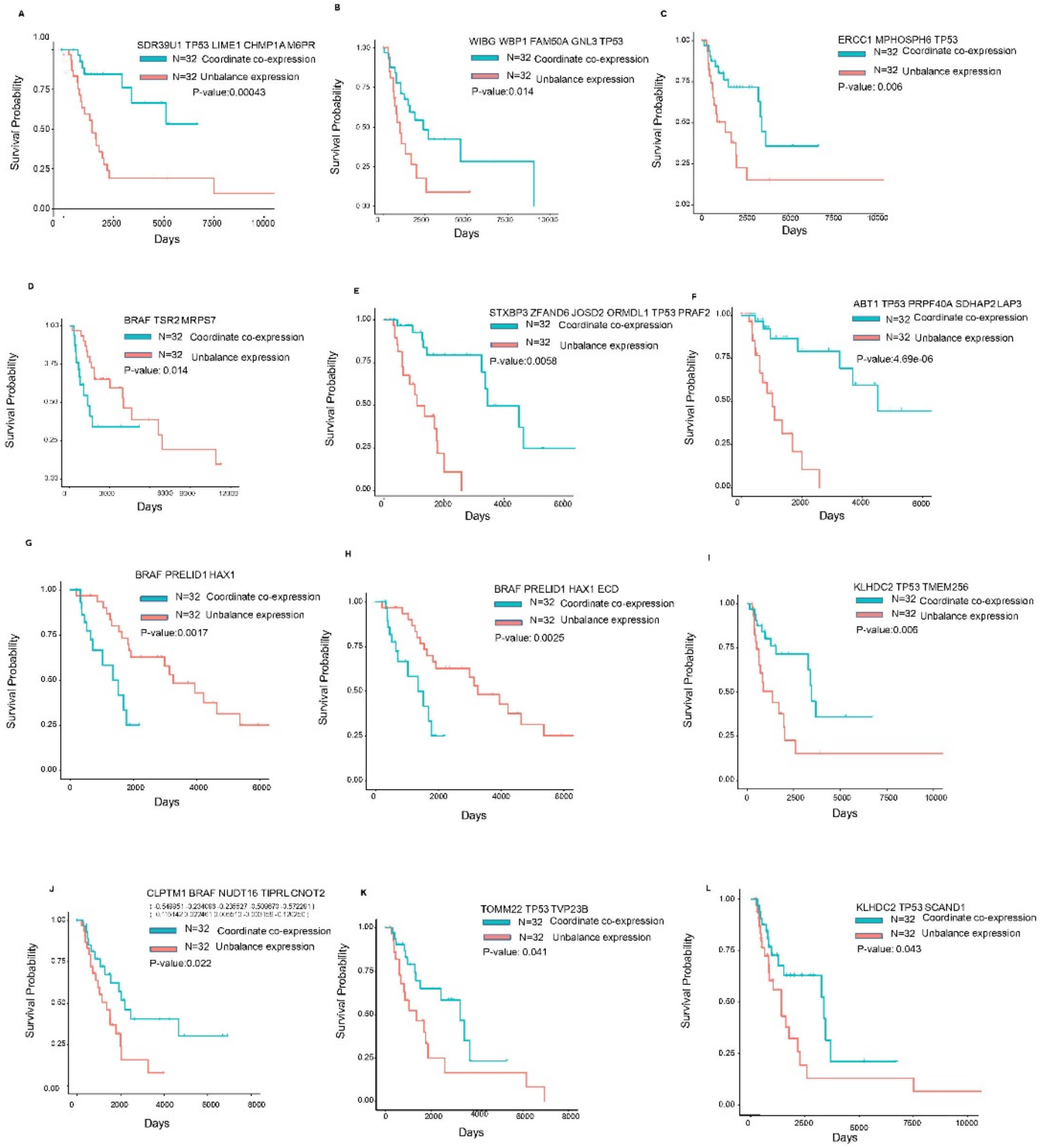

S2 Fig. Survival analysis for top 12 selected COAC-inferred gene co-expression subnetworks from scRNA-seq data in Melanoma patients. The top selected subnetwork for each survival analysis was highlighted in each subfigure. The bulk RNA-seq data and clinical profiles for each melanoma patients were collected from TCGA website [2]. Survival analysis was conducted for these two groups using the R survival package [3].
