# Supplementary material for: A component overlapping attribute clustering (COAC) algorithm for single-cell RNA sequencing data analysis and potential pathobiological implications
Source: PLoS Comput Biol. 2019 Feb 19;15(2):e1006772. doi: 10.1371/journal.pcbi.1006772 (PMC6396937; doi:10.1371/journal.pcbi.1006772)
Supplement: S3 Fig — (PDF) [file pcbi.1006772.s004.pdf]

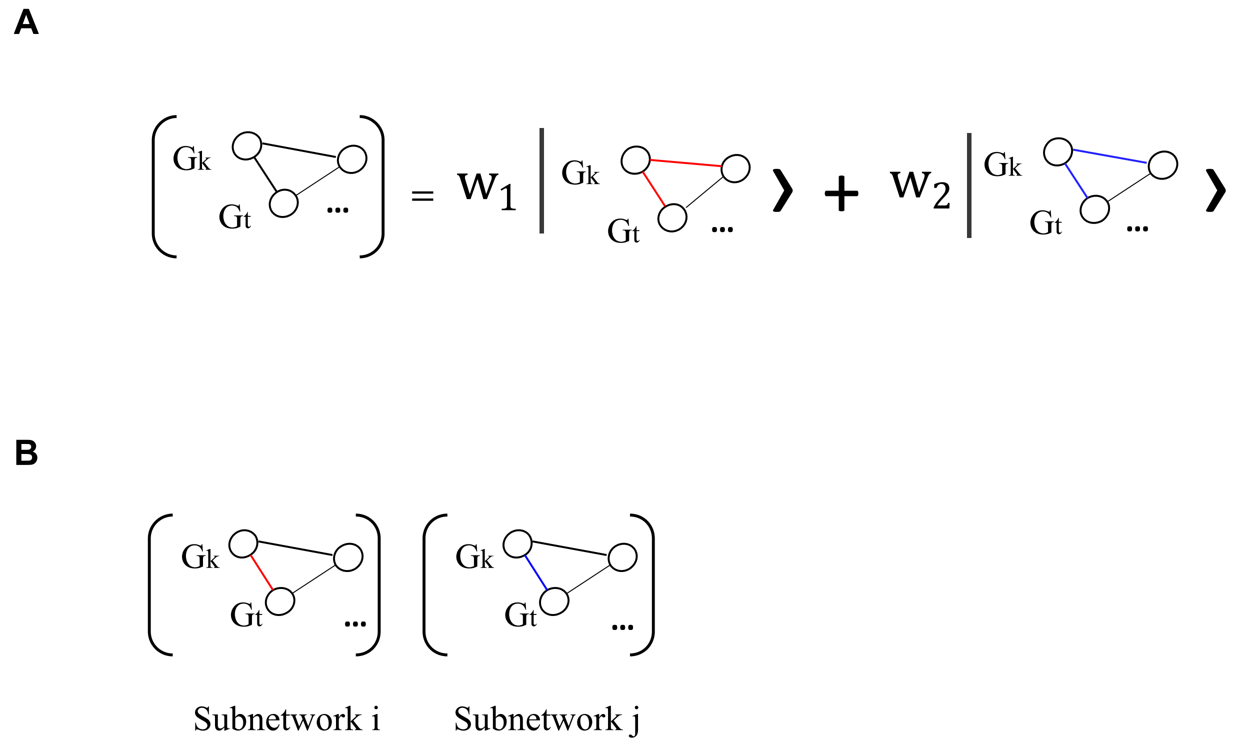

S3 Fig. A diagram illustrating the process of gene co-expression subnetwork identification by COAC. (**A**) Each co-expression sub-network can be treated as a superposition of two different gene expression state. (**B**) The gene co-expression relationship can be represented by different subnetworks.
