# Supplementary material for: A component overlapping attribute clustering (COAC) algorithm for single-cell RNA sequencing data analysis and potential pathobiological implications
Source: PLoS Comput Biol. 2019 Feb 19;15(2):e1006772. doi: 10.1371/journal.pcbi.1006772 (PMC6396937; doi:10.1371/journal.pcbi.1006772)
Supplement: S4 Fig — (PDF) [file pcbi.1006772.s005.pdf]

A

The projection of each attribute (gene) in each component.

$\frac{D_n{}^t P_j}{||D_n||}$ The attribute n distribution projected in j component.

	Attrib-1	Attrib-2		Attrib-n	…
P1	$\frac{D_1{}^t P_1}{\|\|D_1\|\|}$	$\frac{D_2{}^t P_1}{\|\|D_2\|\|}$	…	$\frac{D_n{}^t P_1}{\|\|D_n\|\|}$	…
P2	$\frac{D_1{}^t P_2}{\|\|D_1\|\|}$	$\frac{D_2{}^t P_2}{\|\|D_2\|\|}$	…	$\frac{D_n{}^t P_2}{\|\|D_n\|\|}$	…
…	…	…	…	…	…
Pj	$\frac{D_1{}^t P_n}{\|\|D_1\|\|}$	$\frac{D_2{}^t P_n}{\|\|D_2\|\|}$	…	$\frac{D_n{}^t P_j}{\|\|D_n\|\|}$	…
…	…	…	…	…	…

B

The original sparse matrix after rule mapping.

	Attrib-1	Attrib-2		Attrib-n	…
…	…	…	…	…	…
Pj	1	0	…	-1	…
…	…	…	…	…	…

C

-1 is replaced by1 via increasing additional column.

	Attrib-1(P)	Attrib-2(P)		Attrib-n(P)	…	Attrib-1(N)	Attrib-2(N)	…	Attrib-n(N)	
…	…	…	…	…	…	…	…	…	…	…
Pj	1	0	…	0	…	0	0		1	…
…	…	…	…	…	…	…	…	…	…	…

D

		X	Y	Z	
	…	…	…	…	…
M	…	1	1	1	…
N	…	1	1	1	…
	…	…	…	…	…

E

Associate rule {X Y Z} {M N}

S4 Fig. A diagram illustrating matrix factorization method for gene co-expression subnetwork identification. (**A** and **B**) The projection of each attribute distribution over each principal component distribution. (**C**) A binary matrix composed of 1 or 0 after rule mapping. (**D** and **E**) Associate rules were obtained from the sub-tables of binary matrix whose all elements are no-zero. For the domain of "-1", we translated "–1" to "1" by increasing an additional column.
