# Supplementary material for: A component overlapping attribute clustering (COAC) algorithm for single-cell RNA sequencing data analysis and potential pathobiological implications
Source: PLoS Comput Biol. 2019 Feb 19;15(2):e1006772. doi: 10.1371/journal.pcbi.1006772 (PMC6396937; doi:10.1371/journal.pcbi.1006772)
Supplement: S5 Fig — (PDF) [file pcbi.1006772.s006.pdf]

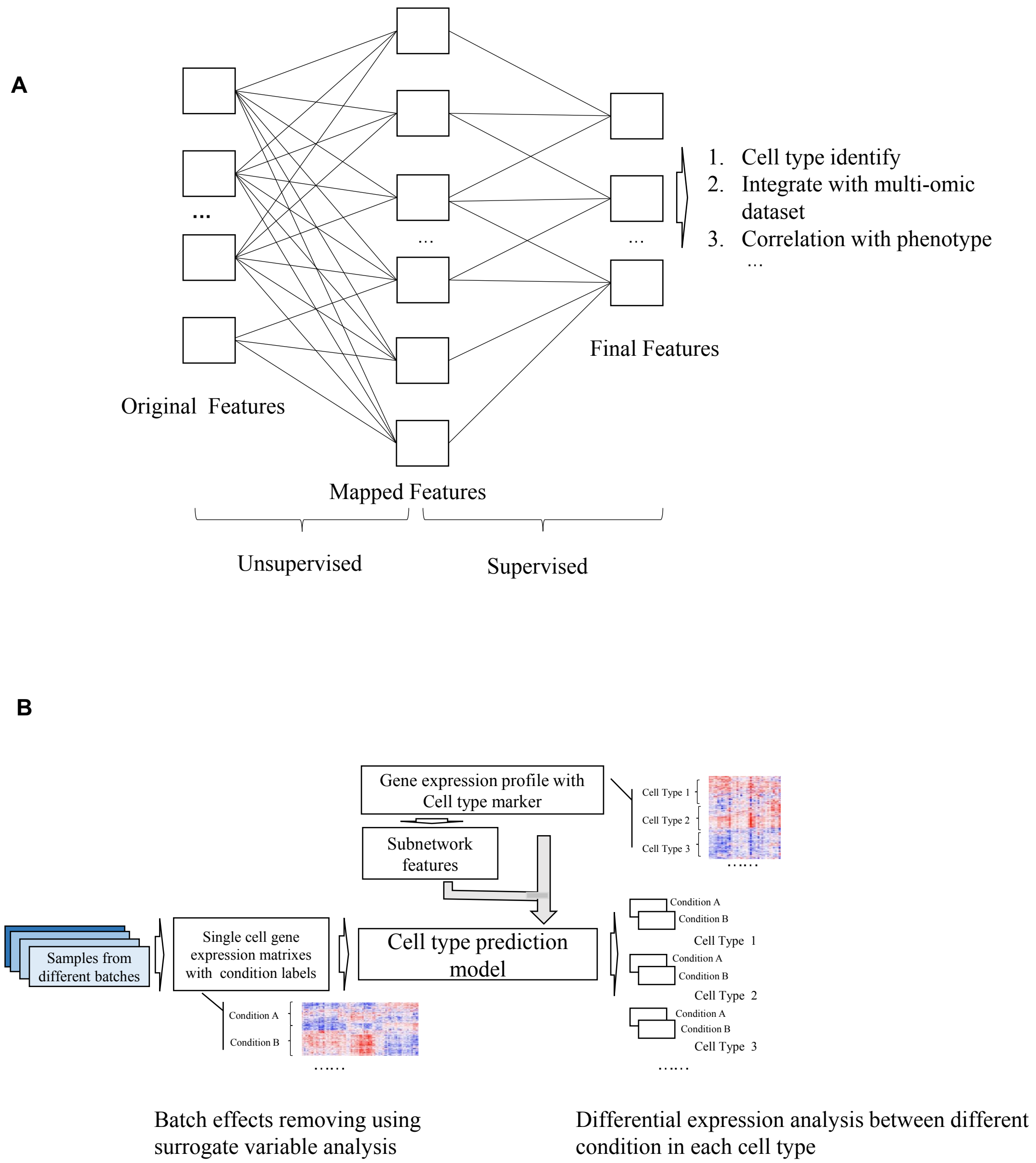

S5 Fig. A diagram illustrating of the pipeline of cell type identification by COAC. (**A**) A diagram shows the pipeline from single gene to gene co-expression network features and the final features will be obtained from gene co-expression subnetworks in a supervised way. (**B**) A pipeline illustrating for cell type identification.
