# Supplementary figures and images for: A component overlapping attribute clustering (COAC) algorithm for single-cell RNA sequencing data analysis and potential pathobiological implications
Source: PLoS Comput Biol. 2019 Feb 19;15(2):e1006772. doi: 10.1371/journal.pcbi.1006772 (PMC6396937; doi:10.1371/journal.pcbi.1006772)

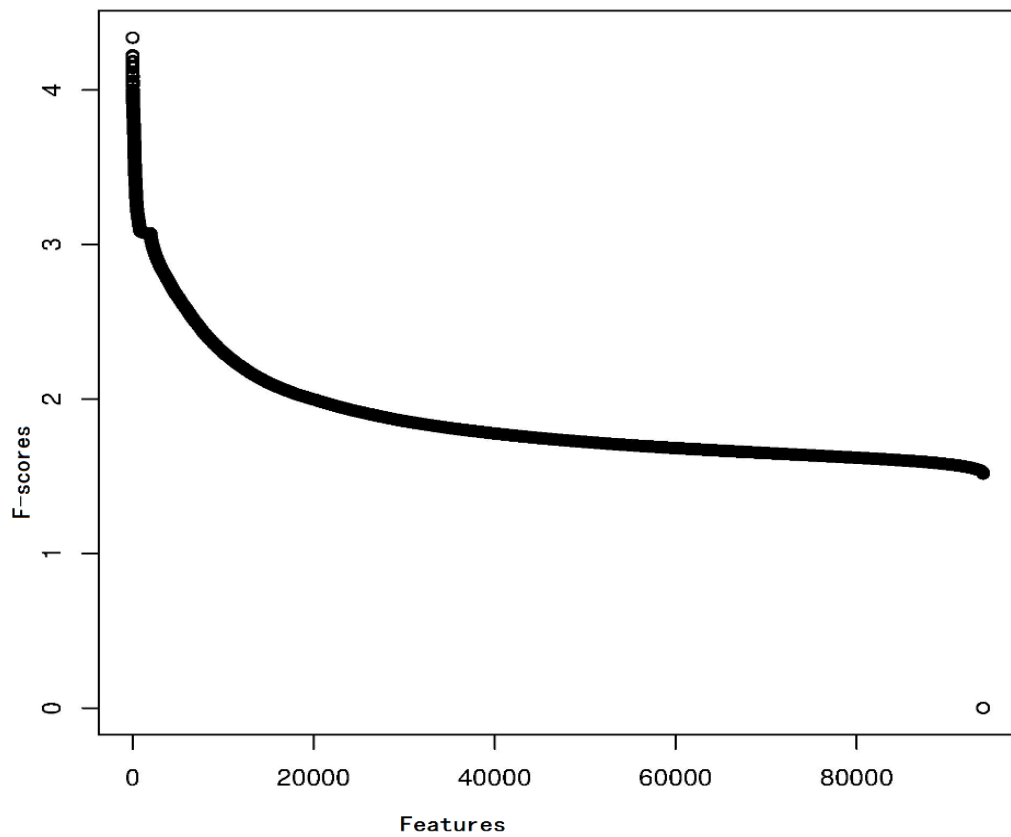

**S6 Fig. Distribution of the ratio (F-score) of the differential variance and background variance.**

Supplement: S6 Fig — (PDF) [file pcbi.1006772.s007.pdf]
