# Supplementary material for: A component overlapping attribute clustering (COAC) algorithm for single-cell RNA sequencing data analysis and potential pathobiological implications
Source: PLoS Comput Biol. 2019 Feb 19;15(2):e1006772. doi: 10.1371/journal.pcbi.1006772 (PMC6396937; doi:10.1371/journal.pcbi.1006772)
Supplement: S7 Fig — (PDF) [file pcbi.1006772.s008.pdf]

Binary distribution matrix

Attribute Name / Component ID	RMND1	ANKRD54	MC2R
2015	1	1	1
641	1	1	1
281	X	X	X
609	X	X	X
899	X	X	X
1414	X	X	X
...	...	...	...

X means it is 1/0 , not always 1

Principle components contribution in each attributes

Attribute Name / Component ID	RMND1	ANKRD54	MC2R
2015	-4.012	-4.903	-1.069
641	4.436	3.949	0.872
281	1.093	1.369	0.273
609	1.261	1.342	0.286
899	-1.577	-1.688	-0.396
1414	-1.238	-1.115	-0.269
...	….	….	….

Rows 2015 and 641: The original components in this closed associate rule which have significant value in binary distribution matrix.

Rows 281 to ...: The components which have similar pattern with original components.

S7 Fig. A diagram illustrating the processes of binary distribution matrix analysis and principle components contribution analysis. The selected components containing all components whose distribution is similar with the components in the component collection of this closed associate rule.

References

1. Tirosh I, Izar B, Prakadan SM, Wadsworth MH, Treacy D, Trombetta JJ, et al. Dissecting the multicellular ecosystem of metastatic melanoma by single-cell RNA-seq. Science. 2016; 352(6282):189-96.

2. Bewick V, Cheek L, Ball J. Statistics review 12: survival analysis. Crit. Care. 2004; 8(5):389.

3. Therneau T, Lumley T. Survival: Survival analysis, including penalised likelihood. R package version 2.35-7. R foundation for Statistical Computing 2011.
